# Weight stigma in maternity care: women’s experiences and care providers’ attitudes

**DOI:** 10.1186/1471-2393-13-19

**Published:** 2013-01-22

**Authors:** Kate Mulherin, Yvette D Miller, Fiona Kate Barlow, Phillippa C Diedrichs, Rachel Thompson

**Affiliations:** 1 The University of Queensland, Queensland Centre for Mothers and Babies, School of Psychology, Brisbane, Australia; 2 Queensland University of Technology, School of Public Health and Social Work, Brisbane, Australia; 3 The University of Queensland, School of Psychology, Brisbane, Australia; 4 Centre for Appearance Research, University of the West of England, Bristol, UK; 5 The Dartmouth Center for Health Care Delivery Science, Dartmouth College, Hanover, USA

**Keywords:** Weight stigma, Maternity care, Prejudice, Obesity, Pregnancy

## Abstract

**Background:**

Weight stigma is pervasive in Western society and in healthcare settings, and has a negative impact on victims’ psychological and physical health. In the context of an increasing focus on the management of overweight and obese women during and after pregnancy in research and clinical practice, the current studies aimed to examine the presence of weight stigma in maternity care. Addressing previous limitations in the weight stigma literature, this paper quantitatively explores the presence of weight stigma from both patient and care provider perspectives.

**Methods:**

Study One investigated associations between pre-pregnancy body mass index (BMI) and experiences of maternity care from a state-wide, self-reported survey of 627 Australian women who gave birth in 2009. Study Two involved administration of an online survey to 248 Australian pre-service medical and maternity care providers, to investigate their perceptions of, and attitudes towards, providing care for pregnant patients of differing body sizes. Both studies used linear regression analyses.

**Results:**

Women with a higher BMI were more likely to report negative experiences of care during pregnancy and after birth, compared to lower weight women. Pre-service maternity care providers perceived overweight and obese women as having poorer self-management behaviours, and reported less positive attitudes towards caring for overweight or obese pregnant women, than normal-weight pregnant women. Even care providers who reported few weight stigmatising attitudes responded less positively to overweight and obese pregnant women.

**Conclusions:**

Overall, these results provide preliminary evidence that weight stigma is present in maternity care settings in Australia. They suggest a need for further research into the nature and consequences of weight stigma in maternity care, and for the inclusion of strategies to recognise and combat weight stigma in maternity care professionals’ training.

## Background

Weight stigma is the exhibition of prejudiced attitudes (e.g., attribution of negative labels such as lazy, unclean, and unintelligent) and discriminatory actions (e.g., teasing, providing inferior quality education, health or other services) towards an individual based upon their weight and body size alone (see [1] for a review). Weight stigma is the fourth most common form of discrimination in the United States, and studies from Australia, Europe and North America document its presence across a range of professional settings [2]. Weight stigma has serious negative consequences for targeted individuals’ psychological and physical health. These include poor psychological functioning [3], body dissatisfaction [4], increased episodes of binge-eating [5,6] and exercise avoidance [7], even after controlling for the direct effect of actual body size. People who report experiencing weight stigma in healthcare settings are more likely to delay medical appointments and preventive healthcare procedures [8].

Despite its associated negative health outcomes, a large number of studies have documented weight stigma in healthcare settings. Healthcare providers respond differentially to patients on the basis of their body size [9]. Stigmatisation of overweight and obese people has been observed among doctors, nurses, psychologists, obesity specialists and pre-service health students [10]. One study found that physicians are likely to assume that overweight or obese patients are less healthy, less self-disciplined and take poorer care of themselves than lower-weight patients [11]. The same study showed that physicians report being more annoyed and experiencing less work enjoyment when treating obese patients.

The prevalence of weight stigma in the maternity care sector has been relatively unexamined. This is an important gap, given an increased focus on both the risks and management of maternal overweight and obesity during pregnancy in recent years. A burgeoning literature is concerned with risks associated with obesity in pregnancy [12,13]. This increased scientific focus has been accompanied by the recent emergence of clinical guidelines on the management of obesity in pregnancy [14,15] and increased attention to obesity in pregnancy in the media [16]. Greater preoccupation with obesity in the maternity care sector may have increased the salience of a woman’s body shape and size for maternity care providers and thus increased the likelihood of weight stigma occurring in the provision of maternity care [17].

The potential for weight stigma in maternity care is of particular concern, given that women are at increased risk for depression and other mental health issues during pregnancy and the puerperium [18]. Given the demonstrated negative impact of weight stigma experiences on mental health, it is possible that such experiences in maternity care may increase the risk of psychological difficulties during a period of already increased vulnerability. However, relatively few studies have examined the presence of weight stigma in maternity care settings.

Three recent qualitative studies found stigmatising attitudes among maternity care providers, including high levels of discomfort, intolerance, and feelings of repulsion in caring for obese pregnant women [19,20], as well as the assumption that obese women lacked necessary skills, awareness, or motivation to manage their weight [21]. Together, these findings suggest that care providers’ attitudes were stigmatising and ultimately unhelpful. The exact prevalence of weight stigma in the maternity care sector, however, remains unknown. In particular, it is unclear whether discriminatory attitudes and behaviours are widespread in the maternity care sector or, instead, whether incidents of discrimination are perpetrated by a small group of care providers with a specific anti-fat bias. Accordingly, there is little evidence for determining if calls for anti-weight stigma initiatives in other health professions e.g., [22] should be extended to maternity care settings.

Qualitative studies also suggest that overweight and obese maternity care consumers perceive discrimination on the basis of their weight. These studies found that obese women reported instances of negative interactions with maternity care providers, including perceptions that care providers were rude, angry and abrupt, did not take them seriously, and did not adequately respond to their needs [23,24]. Women report feeling embarrassed, isolated, receiving insufficient and/or inconsistent information about appropriate engagement in health behaviours in pregnancy, and desiring greater support from health professionals throughout the perinatal period [21,25]. The women in these studies attributed their perceived negative treatment from care providers to their ‘larger–than-normal’ body size and weight, providing preliminary support for the existence of weight stigma in the maternity care sector. Overall, however, these studies failed to include a normal-weight comparison group of pregnant women, precluding definitive conclusions that perceptions of poor quality care are actually higher among individuals who are overweight and obese, than among normal-weight individuals. Further, these studies were conducted in the UK [21,23,25] and Sweden [24]; it is important, then, to investigate whether experiences of weight stigma in maternity care are also evident among women in the Australian context, where obesity is increasingly on the agenda in maternity healthcare settings [12,15].

Notably, two recent studies have sought to incorporate a normal-weight comparison group to investigate weight stigma in maternity care, though these have been limited to investigating perspectives of pregnant women, without looking at maternity care providers’ attitudes. A recent Australian large-scale survey explicitly investigating women’s experiences of discrimination in maternity care found an association between obesity in pregnancy and perceived discrimination [26]. A recent Swedish survey found that women with a BMI above 30 reported a less positive experience of pregnancy, and experienced less continuity of care, than women with a lower BMI [27]. However, women were quite satisfied with the care they received, with no differences observed on the basis of BMI. Hildingsson and Thomas [27] hypothesised that this may be due to midwives’ reluctance to raise weight issues throughout pregnancy care, leading to a generally positive experience among higher-BMI women who desire the focus in their maternity care to be placed on their pregnancies and births, and not their weight. The authors highlighted a need to investigate any differences in health professionals’ provision of maternity care to women of differing body size.

Overall, our understanding of the nature and extent of weight stigma in the maternity care sector is limited by the relatively small number of studies investigating weight stigma from women’s and care providers’ perspectives, the qualitative focus of the majority of studies, and design limitations of some of this research. This paper comprises the first quantitative investigation of weight stigma in maternity care, from the perspectives of *both* women and care providers. It describes two complementary studies to examine the existence of weight stigma in maternity care in Australia from the perspective of normal-, overweight and obese pregnant women, and pre-service medical and midwifery maternity care providers.

Study One examined the relationship between a woman’s self-reported pre-pregnancy body mass index (BMI) and perceived quality of her treatment by care providers during and after pregnancy, using a large sample of women who had recently given birth in Queensland, Australia. In this study, quality of treatment focused largely on the quality of interpersonal treatment from care providers (including a sense of being listened to and understood; being treated with respect), which is increasingly regarded as an important aspect of women’s experience of maternity care [28]. Importantly, this study used two measures of perceived quality of treatment from care providers: perceived positive treatment (assessing women’s general experiences of feeling respected and cared for) and perceived negative treatment (assessing women’s experiences of instances where they were not treated with respect, care or understanding). In light of evidence that higher BMI is associated with more negative treatment in general healthcare settings [11,29], it was expected that women with a higher pre-pregnancy BMI would perceive less positive and more negative treatment from maternity care providers.

Study Two examined the effect of a hypothetical pregnant woman’s pre-pregnancy BMI on the attitudes of pre-service medical and midwifery maternity care providers^a^ towards the woman, and perceptions of her self-management behaviours and health. A student sample was used to gain an understanding of attitudes among maternity care professionals of the future, and to identify any need for intervention during formative stages of training. Pre-pregnancy BMI was experimentally manipulated in case management test cases to assess its effect on pre-service care providers’ perceptions of, and attitudes towards, caring for the patient. We hypothesised that pre-service care providers would report more negative perceptions and attitudes towards women with an overweight and obese BMI, compared to women with a normal-weight BMI.

We were also interested in whether any effect of pre-pregnancy BMI on care providers’ perceptions and attitudes was moderated by care providers’ more general weight stigmatising attitudes (i.e., ‘anti-fat’ attitudes not specific to maternity care settings), to establish whether incidences of weight stigma in maternity care settings were only evident among care providers with high general weight stigmatising attitudes. We hypothesised that an association between negative attitudes/perceptions and higher patient BMI would be strongest among pre-service care providers with higher levels of general weight stigma, compared to those with lower levels. Finally, we also explored whether any effects of pre-pregnancy BMI on care providers’ perceptions and attitudes were moderated by the BMI of the care providers themselves.

## Methods

### Sampling and recruitment

#### Study one

Study One analysed data collected from the “Having a Baby in Queensland” Pilot Survey, conducted by the Queensland Centre for Mothers and Babies (QCMB) in November 2009. The QCMB partnered with the Queensland Registry of Births, Deaths and Marriages (BDM) to recruit participants, based on formal notifications by care providers of all births in Queensland in a two-week period. Women were eligible for participation in the survey if they had a single, live born baby during this period and a complete address recorded (*n* = 2306).

Eligible women were sent a survey invitation package when their infants were approximately three months old. The invitation package contained a paper version of the survey, a reply-paid envelope and a fridge magnet as a gift. Two weeks after the first package was sent, a follow-up package was posted, regardless of whether women had responded. Confidential sampling via the Registry of Births, Deaths and Marriages meant we were unable to identify, and send tailored reminders to, non-responders specifically. Women were able to complete the survey on paper (and return using the reply-paid envelope), online or over the telephone (by free call with trained interviewers or in any language with an interpreter). In total, 2240 survey packages were delivered (66 packages were returned undelivered) and 693 women responded to the survey, yielding a response rate of 31%. Of the 693 survey respondents, 627 provided sufficient information about their pre-pregnancy weight and height to allow for the calculation of their pre-pregnancy BMI.

The survey and collection methods were approved by the Behavioural and Social Sciences Ethical Review Committee at The University of Queensland (Ethics Clearance #2009001531).

#### Study two

All major institutions throughout Australia offering a medical degree and/or an undergraduate midwifery degree were invited to recruit participants. Administrators at these institutions were asked to forward an email from the researcher to eligible students, inviting them to participate in an online survey. Participants were offered entry into a draw to win a $100 shopping voucher in exchange for participation.

Of 18 medical schools contacted, six consented to inviting their students to participate. Approximately 2200 medical students were invited to participate, and 215 completed the survey, yielding a response rate of about 10%. Of nine midwifery departments, three consented to invite their students, and surveys were administered in person to 22 midwifery students from one institution. Students from the other two institutions completed the survey online. Of 55 midwifery students invited to participate overall, 33 responded, yielding a response rate of about 60%.

The study and collection procedure were approved by a subcommittee of Behavioural and Social Sciences Ethical Review Committee at The University of Queensland (approval # 10-PSYCH-4-114-JM).

### Participants

#### Study one

Participants were 627 women who gave birth in Queensland in 2009.

#### Study two

Participants were 248 pre-service maternity care providers in Australia, comprising 215 medical students from six universities, and 33 midwifery students from three universities. Medical students were in the final two years of their medical degree, and had completed, or were currently completing, a General Practice rotation^b^. All midwifery students were in their final (third) year of an undergraduate midwifery degree.

### Measures

#### Study one

The “Having a Baby in Queensland” survey employed a cross-sectional design, and included retrospective self-reported measures of the quality of maternity care provided, information and support provided, health outcomes, and socio-demographic variables. Measures relevant to the current study are detailed below.

### Pre-pregnancy body size

Pre-pregnancy BMI was calculated by dividing participants’ self-reported pre-pregnancy weight in kilograms by the square of their height in metres (weight (kgs)/height (m)^2^).

### Socio-demographic characteristics

Participants’ reported highest level of education was coded from 1 (No school) to 12 (Higher University Degree); a higher score indicated a higher education level. Country of birth was also assessed *(“In what country were you born?”*). Women’s age at birth was calculated from participants’ reported date of birth, and the date of their baby’s birth. Participants reported their infant’s gestational age at birth *(“How many weeks pregnant were you when your baby was born?*”). The approximate age of each participant’s baby at survey completion was calculated from the reported date of their baby’s birth and date of survey receipt.

### Perceived quality of treatment

Participants responded to a set of statements assessing separately their perceived quality of treatment during pregnancy, during labour and birth, and after birth. Participants were asked, *“Thinking about your care during your pregnancy/during your labour and birth/after having your baby, how much do you agree or disagree with the following statements?”*, and responded to the same set of statements in reference to each time period. The statements, each of which utilised a Likert response format (from 1 = “strongly disagree” to 5 = “strongly agree”) formed two scales reflecting participants’ *“perceived positive treatment”* and *“perceived negative treatment”*.

Perceived positive treatment was derived by averaging participant responses to the following four statements: *“My carers treated me with respect”*, *“My carers treated me with kindness and understanding”*, *“My carers respected my privacy”*, and *“My carers genuinely cared about my wellbeing”*, with higher scores indicating higher perceived positive treatment. The scale demonstrated high internal consistency for each time period (*α*s > .89).

Perceived negative treatment was derived by averaging participant responses to the following four statements: “*One or more of my carers did not treat me with respect”*, *“One or more of my carers did not treat me with kindness and understanding”*, *“One or more of my carers were not open and honest”*, and *“One or more of my carers did not genuinely care about my wellbeing”*, with higher scores indicating more perceived negative treatment. The scale demonstrated high internal consistency for each time period (αs > .90*)*

#### Study two

A novel survey was developed to assess care providers’ attitudes towards, and perceptions of, normal-weight, overweight and obese pregnant women. Participants first read a hypothetical patient case about a pregnant woman who was either normal-weight, overweight or obese. They were then asked questions about perceptions of, and attitudes towards, caring for the patient described. The survey was administered online to the majority of participants, and randomly generated either the normal-weight, overweight or obese patient case for each participant. The second half of the survey included demographic questions and a measure of weight stigmatising attitudes.

### Development of patient case presentation

Participants were presented with a hypothetical case detailing a pregnant woman presenting for an initial appointment in pregnancy, with either a general practitioner (for medical students) or a midwife (for midwifery students). The BMI of the hypothetical patient was manipulated in case presentations such that participants were randomly allocated to read about a pregnant patient with either a normal-weight BMI, overweight BMI, or obese BMI. Descriptions of the patient’s appearance in terms of weight (e.g., “Debbie appears to be overweight”) as well as measures of height, weight and BMI were provided to ensure effective manipulation of the independent variable. A range of information was included to enhance ecological validity and to indicate that the patients were equal on all other indices of health, and were not at risk for other complications. This information was kept consistent across all conditions.

### Perceptions of patient self-management and health

Perceptions of patient self-management and health was measured using a 3-item scale, adapted from Hebl and Xu [11], with a Likert response format from 1 (“Highly Unlikely”) to 7 (“Highly Likely”). Items included *“Overall, the patient is healthy”*, *“Overall, the patient takes care of herself”*, and *“The patient is self-disciplined”.* Scores for the scale were calculated by averaging participants’ scores across items. A higher score was associated with more positive perception of the patient’s self-management and health. The scale demonstrated high internal consistency with the current sample (α= .88).

### Attitudes towards caring for patient

Attitudes towards caring for the patient were measured via a 6-item scale adapted from Hebl and Xu [11], which used the same response format and scoring methods as above. Scale items were *“This sort of patient would make me like my job”*, *“I would have a lot of patience with this patient”*, *“This patient would annoy me”*, *“I would have a significant personal desire to help and support this patient”*, *“Overall, I would feel positive towards this patient”*, *and “Seeing this patient would feel like a waste of my time”.* Scores for the scale were calculated by averaging participants’ scores across items, with the two items endorsing a negative attitude reverse-scored. A higher score was associated with a more positive attitude towards caring for the patient. The scale had high internal consistency in this sample (α= .86).

### Socio-demographic characteristics

Gender and age were self-reported.

### Body size

Participants were asked to report their own height and weight. A continuous measure of participant BMI was calculated, as in Study One.

### Weight stigmatising attitudes

Participants’ weight stigma attitudes were assessed using Lewis et al.’s [30] Anti-Fat Attitudes Test (AFAT). The AFAT provides a measure of an individual’s general level of weight stigmatising attitudes and beliefs, incorporating the extent to which individuals attribute negative characteristics and stereotypes to overweight and obese people. The scale contains items indicative of anti-fat attitudes (e.g., *“I’d lose respect for a friend who started getting fat”*; *“Most fat people are lazy”),* with response options from 1 (“definitely disagree”) to 5 (“definitely agree”). Total scale scores were calculated by averaging responses across all items. Six items endorsing positive or neutral attitudes (e.g. *“If I were single, I would date a fat person”)* were reverse-scored. Overall, higher scores indicated higher weight stigmatising attitudes. The scale had high internal consistency (α= .91).

### Procedure

#### Study one

### Statistical analyses

T-tests and chi-square tests were conducted to determine differences between women who did not report their weight and/or height (excluded from the sample) and women in the final sample. Six hierarchical linear regression analyses were conducted to examine relationships between pre-pregnancy BMI and the dependent variables (perceived positive and negative quality of treatment from care providers in pregnancy, labour and birth, and after birth). Past research has found that pre-pregnancy BMI is negatively related to education [12], so all analyses routinely controlled for education to ensure that any observed effects were attributable to BMI. In each regression analysis, maternal education level was entered in Block 1, followed by pre-pregnancy BMI in Block 2. Significance was set to *p* < 0.05 for all analyses. Notably, only a very small number of women (<1%) had a BMI in the underweight range (BMI < 18.5), which precluded any separate analysis of this group.

#### Study two

### Surveys administered in person

Surveys were administered in person to 22 midwifery students. Surveys were handed out in such an order that participants were randomly assigned to one of the three BMI conditions. Participants were first given the patient case and all measures except the AFAT. Participants were given the AFAT only after completion and collection of the first part of the survey, to prevent potential response bias for earlier survey questions due to exposure to the AFAT.

### Surveys administered online

Participants accessed the online survey via a link in the invitation email. One of three patient cases (varying on patient BMI) was then randomly presented to participants, followed by the survey measures. Participants were unable to return to previous survey pages, to prevent bias associated with awareness of the experimental manipulation when they were presented with the AFAT.

### Statistical analyses

Chi-square tests and one-way Analyses of Variance were conducted to assess differences between experimental groups on demographic characteristics and AFAT scores. The effect of patient BMI on pre-service care providers’ perceptions of, and attitudes towards caring for, the patient, as well as potential moderating effects of anti-fat attitudes and care providers’ BMI, were investigated with two hierarchical moderated regression analyses. In the first regression, perceptions of patient self-management and health was the criterion variable, and in the second, attitudes towards caring for the patient was the criterion variable. Using unweighted effect coding, two patient BMI contrasts were created to allow for the comparison of perceptions and attitudes between the different patient BMI conditions. The first variable contrasted the normal-weight patient BMI condition to those in the overweight and obese conditions. The second variable contrasted the overweight patient BMI condition to the obese patient BMI condition. Mean-centred AFAT scores and participant BMI were entered at Step 1 of the regression analyses, followed by the two patient BMI contrasts entered at Step 2. To explore the moderating effects of anti-fat attitudes, two interaction terms for each of the patient BMI condition contrasts and anti-fat attitudes (AFAT) scores were entered at Step 3. Additionally, to explore any moderating effects of participant BMI, two interaction terms for each of the patient BMI condition contrasts and participant BMI were entered at Step 3. Power analyses indicated that 159 participants were required to have an 80% chance of detecting medium effect sizes [31], thus the analyses were collapsed across medical and midwifery pre-service care providers to maximise power.

## Results

### Study one

#### Sample characteristics

Participants were 627 women with a mean pre-pregnancy BMI of 24.66 kg/m^2^ (*SD =* 5.14 kg/m^2^, range = 15.57 – 46.50 kg/m^2^). Participants’ mean age at time of their most recent birth was 30.02 years (*SD =* 5.21 years, range = 15.00 – 43.00 years), and their infants’ mean gestational age at birth was 39.27 weeks (*SD =* 2.09 weeks, range = 21.60 – 44.00 weeks). The majority of women were born in Australia (79.7%), and a very small proportion (1.8%) was of Aboriginal or Torres Strait Islander status. Approximately 48% of women in the sample were primiparous (i.e., their most recent birth was their first). A substantial proportion of women had completed a Bachelor degree or higher (40%), although 22% had completed education no further than secondary school.

There were no significant differences in maternal and infant gestational age at birth between women who were excluded due to missing height/weight data (N = 66) and women in the final sample (N = 627). Compared to women in the final sample, women with no BMI data were significantly more likely to be multiparous (i.e., have given birth more than once; 63.6% vs. 51.4%, χ^2^ (1) = 4.53, *p* = .033) and less likely to have completed a Bachelor degree or higher (27.3% vs. 40.0%, χ^2^ (2) = 8.56, *p* = .014). There were no significant differences between women excluded and women included in the final sample on any dependent variables in this study.

### Perceived quality of treatment during pregnancy

#### Perceived positive treatment

In Block 1, education did not account for a significant amount of variance in perceived positive treatment during pregnancy, *R*^*2*^ = .006, *F*(1, 618) = 3.63, *p* = .057. In Block 2, pre-pregnancy BMI did not significantly contribute to variance in perceived positive treatment during pregnancy [*R*^*2*^ = .006, *R*^*2*^_change_ = .000, *F*_change_(1, 617) = .03, *p* = .865], and the overall model was not significant, *F*(2, 617) = 1.83, *p* = .162.

#### Perceived negative treatment

In Block 1, education did not account for a significant amount of variance in perceived negative treatment during pregnancy *R*^*2*^ = .004, *F*(1, 613) = 2.69, *p* = .101. With the addition of pre-pregnancy BMI in Block 2, the model became significant, *R*^*2*^ = .019, *F*(2, 612) = 5.86, *p* = .003, *R*^*2*^_change_ = .014, *F*_change_(1, 612) = 8.99, *p* = .003. Pre-pregnancy BMI was a significant predictor of perceived negative treatment in pregnancy (*β* = .12, *p* = .003), such that women with a higher BMI perceived more negative treatment during pregnancy.

### Perceived quality of treatment: labour and birth

#### Perceived positive treatment

In Block 1, education did not account for a significant amount of variance in perceived positive treatment during labour and birth, *R*^2^ = .004, *F*(1, 615) = 2.58, *p* = .109. The addition of pre-pregnancy BMI in Block 2 did not account for any additional variance [*R*^2^_ change_ = .001, *F*_ change_(1, 614) = .42, *p* = .516], and the overall model was not significant, *R*^2^ = .005, *F*(2, 614) = 1.50, *p* = .224. Therefore, there was no effect of pre-pregnancy BMI on perceived positive treatment during labour and birth.

#### Perceived negative treatment

In Block 1, education did not account for a significant amount of variance in perceived negative treatment during labour and birth, *R*^2^ *=* .000, *F*(1, 605) = .24, *p* = .625. In Block 2, pre-pregnancy BMI did not account for any additional variance [*R*^2^_change_ = .001, *F*_change_(1, 604) = .65, *p* = .419], and the overall model was not significant, *R*^2^ *=* .001, *F*(2, 604) = .45, *p* = .640. Therefore, there was no effect of pre-pregnancy BMI on perceived negative treatment during labour and birth.

### Perceived quality of treatment: after birth

#### Perceived positive treatment

In Block 1, education did not account for a significant amount of variance in perceived positive treatment after birth, *R*^2^ *=* .000, *F*(1, 611) = .02, *p =* .880. With the addition of pre-pregnancy BMI in Block 2, the model accounted for significant variance in perceived positive treatment, *R*^2=^.011, *F*(2, 610) = 3.39, *p* = .034, *R*^2^_change_ = .011, *F*_change_(1, 610) = 6.73, *p* = .010. Pre-pregnancy BMI significantly predicted positive treatment after birth (*β = −*.11, *p* = .010), such that a higher BMI was associated with less perceived positive treatment.

#### Perceived negative treatment

In Block 1, education did not account for a significant amount of variance in perceived negative treatment after birth, *R*^2^ *=* .005, *F*(1, 601) = 1.80, *p* = .180. With the addition of pre-pregnancy BMI in Block 2, the model remained non-significant, *R*^2^ *=* .004, *F*(2, 600) = 1.26, *p =* .284, *R*^2^_change_ = .001, *F*_change_(1, 600) = .73, *p =* .395. Therefore, there was no effect of pre-pregnancy BMI on perceived negative treatment after birth.

### Study two

#### Sample characteristics

Participants’ mean age was 25.29 years (*SD =* 4.58 years; range = 19–50 years), and the majority of participants (71%) were female. Mean BMI was 22.61 kg/m^2^ (*SD =* 3.38 kg/m^2^; Range = 15.43-41.50 kg/m^2^). Participants’ mean AFAT score was 2.17 (*SD =* .49, Range = 1–3.73), below the scale mid-point. No significant differences were revealed between groups in gender, age, BMI and AFAT scores.

#### Effects of patient BMI

Table 1 displays the means and standard deviations for all dependent variables, for each experimental group.

**Table 1:** Means and Standard Deviations of Perceptions and Attitudes across Patient BMI conditions

	**Patient BMI condition**		
	**Normal (N = 82)**	**Overweight (N = 82)**	**Obese (N = 84)**
*Dependent Variables*			
	Mean (SD)	Mean (SD)	Mean (SD)
Perceptions of Patient Self-Management (1 – 7)	5.54 (.79)	4.69 (1.07)	4.01 (1.10)
Attitudes toward Patient Care (1 – 7)	5.85 (.77)	5.56 (.86)	5.39 (.86)

#### Perceptions of patient self-management and health

Table 2 provides a summary of the regression analysis, including change statistics for each step, and beta coeffi-cients for each predictor in the final model. At Step 1, AFAT level and participant BMI were significantly associated with perceptions of patient self-management and health. The addition of the patient BMI contrasts at Step 2 was significant, indicating that exposure to patient BMI condition accounted for further variance in perceptions of the patient’s self-management and health. The addition of the interaction terms at Step 3 was not significant, indicating that neither weight stigmatising attitudes nor participant BMI significantly moderated the impact of patient BMI on perceptions of patient self-management and health. The final model accounted for a significant proportion of the total variation in perceptions of the patient’s self-management and health (*R*^2^ = .34, *adjusted R*^2^ = .32, *F*(8, 230) = 14.74, *p* = .000).

**Table 2:** Effects of patient BMI and AFAT level on Perceptions of Patient Self-Management and Health

**Step and variable**	***b***	***β***	***t***	***sr***^***2***^	***R***^***2***^	***Adjusted R***^***2***^	***Δ R***^***2***^	***df***	***Δ F***
Step 1									
AFAT	-.35	-.15	-2.62**	-.14	.05	.04	.05	2, 236	5.53**
CBMI	.05	.15	2.64**	.14					
Step 2									
NWOO	.41	.48	8.99***	.48	.34	.33	.29	2, 234	51.86***
OWOB	.34	.24	4.37***	.23					
Step 3		.							
NWOO x	.03	.02	.27	.01	.34	.32	.001	4, 230	.07
AFAT									
OWOB x	-.06	-.02	-.35	-.02					
AFAT									
NWOO x	-.001	-.004	-.07	-.004					
CBMI									
OWOB x	.004	.01	.18	.01					
CBMI									

Notes: Reported *b*, *β*, *t*, and *sr*^*2 *^are from the final model; NWOO = normal weight *vs.* overweight/obese contrast; OWOB = overweight *vs.* obese contrast; CBMI = care provider BMI.

*p <0.05, **p <0.01 and ***p < 0.001.

In the final model, AFAT scores, participant BMI and the two patient BMI condition contrasts remained significantly associated with perceptions of patient self-management and health. Higher levels of weight stigmatising attitudes were associated with less positive perceptions of patient self-management and health, irrespective of the patient’s weight. A higher participant BMI was associated with more positive perceptions of patient self-management and health, again, irrespective of the patient’s weight. The significance of the coefficients for the normal-weight patient BMI condition *vs.* the overweight and obese patient BMI contrasts in the final model indicated a significant difference in perceptions between those exposed to the normal-weight pregnant patient and those exposed to the overweight and obese pregnant patients. Participants who read about a normal-weight pregnant woman (*M* = 5.54) had significantly more positive perceptions of the patient’s self-management and health than those who read about an overweight pregnant woman (*M =* 4.69) and those who read about an obese pregnant woman (*M* = 4.01). Further, the significant coefficient for the overweight patient BMI *vs.* obese patient BMI contrast indicated a significant difference in perceptions between those in the overweight patient BMI and obese patient BMI conditions. Specifically, participants who read about an overweight pregnant woman (*M* = 4.69) displayed significantly more positive perceptions of the patient’s self-management and health compared to those who read about an obese pregnant patient (*M* = 4.01). Non-significant interaction terms indicated that these effects were not qualified by participants’ general weight stigmatising attitudes or their own BMI.

#### Attitudes towards caring for patient

Table 3 provides a summary of the regression analysis, including change statistics for each step, and beta coefficients for each predictor in the final model. At Step 1, AFAT scores were significantly associated with perceptions of patient self-management and health. Participant BMI was not significantly associated with attitudes towards caring for the patient. The addition of the patient BMI contrasts at Step 2 was significant, indicating that exposure to patient BMI condition accounted for further variance in attitudes towards caring for the patient. The addition of the interaction terms at Step 3 was not significant, indicating that neither weight stigmatising attitudes nor participant BMI significantly moderated the impact of patient BMI on attitudes towards caring for the patient. The final model accounted for a significant proportion of the total variation in attitudes towards caring for the patient (*R*^2^ = .20, *adjusted R*^2^ = .18, *F*(8, 231) = 7.43, *p* = .000).

**Table 3:** Effects of patient BMI and AFAT level on Attitudes towards Caring for the Patient

**Step and variable**	***b***	***β***	***t***	***sr***^***2***^	***R***^***2***^	***Adjusted R***^***2***^	***Δ R***^***2***^	***df***	***Δ F***
Step 1									
AFAT	-.59	-.34	−5.59***	-.33	.15	.14	.15	2, 237	20.46***
CBMI	.03	.12	1.86	.11					
Step 2									
NWOO	.13	.21	3.59***	.21	.20	.19	.05	2, 235	7.71***
OWOB	.08	.08	1.36	.08					
Step 3									
NWOO x	.02	.01	.23	.01	.21	.18	.01	4, 231	.36
AFAT									
OWOB x	.04	.02	.31	.02					
AFAT									
NWOO x	-.01	-.07	−1.07	-.06					
CBMI									
OWOB x CBMI	.01	.03	.46	.03					

Notes: Reported *b*, *β*, *t*, and *sr*^*2 *^are from the final model; NWOO = normal weight *vs.* overweight/obese contrast; OWOB = overweight *vs.* obese contrast; CBMI = Care Provider BMI.

*p <0.05, **p <0.01 and ***p < 0.001.

In the final model, AFAT scores and the normal-weight patient BMI *vs.* overweight and obese patient BMI condition contrasts remained significantly associated with attitudes towards caring for patient. Higher levels of general weight stigmatising attitudes were associated with less positive attitudes towards caring for the patient, irrespective of the patient’s weight. There was a significant difference in attitudes towards caring for the patient between those exposed to the normal-weight pregnant patient and those exposed to the overweight and obese pregnant patients. Participants who read about a normal-weight pregnant woman (*M* = 5.85) had significantly more positive attitudes towards caring for the patient than those who read about an overweight pregnant woman (*M =* 5.56) and those who read about an obese pregnant woman (*M* = 5.39). The interaction between this contrast variable and AFAT level was not significant, indicating that general weight stigmatising attitudes did not moderate the impact of patient BMI on attitudes towards caring for the patient. The overweight patient BMI condition *vs.* obese patient BMI condition contrast was not significant, indicating that there was no significant difference in attitudes towards caring for patient between those who read about an overweight pregnant woman and those who read about an obese pregnant woman. The interaction term between this contrast variable and AFAT level was not significant, and there was no significant interaction between participant BMI and patient BMI condition.

## Discussion

This paper describes the first quantitative investigation of weight stigma in maternity care from both mothers’ and maternity care providers’ perspectives, and extends recent qualitative research in the field [19-21,23,24]. As hypothesised, findings from Study One revealed associations between a higher pre-pregnancy BMI and poorer perceived quality of treatment during pregnancy and after birth by patients. Further, in Study Two we found that pre-service care providers hold less positive perceptions of patient self-care of, and attitudes towards caring for, overweight and obese compared to normal-weight, pregnant women. These effects were evident in pre-service care providers with both low and high levels of weight stigmatising attitudes. Together, this preliminary evidence suggests that, like in other healthcare settings, weight stigma is present in maternity care.

Previous qualitative research suggests that overweight and obese women report experiencing sub-optimal treatment in maternity care settings, and attribute this to their larger body size and weight [23,24]. Higher-weight individuals have also been shown to perceive more discrimination in healthcare settings than normal-weight adults in the general population [29]. Consistent with this research, in Study One we found that a higher BMI was significantly associated with a tendency to perceive more negative treatment during pregnancy, and to perceive less positive treatment after birth. Such findings suggest that women of larger body size may be differentially disadvantaged with respect to perceived quality of treatment at certain stages of maternity care, in comparison to normal-weight women.

Pre-pregnancy BMI was not related to perceived quality of treatment measures at all stages of maternity care, however, suggesting that the nature of the relationship between BMI and perceived treatment changes throughout the maternity care experience. During pregnancy, BMI was related to perceived negative treatment, but was not related to perceived positive treatment, suggesting that women with larger bodies may be more likely to experience discrete incidences of negative treatment from one (or possibly more) care providers during pregnancy, but are no less likely to perceive positive treatment from care providers collectively. Maternity care providers may perceive pregnant women with larger body sizes to be at greater risk for complications, and to require extra equipment and intervention [32]. Such perceptions may lead to frustration by care providers and give rise to incidences of negative treatment towards the pregnant woman. Possible incidences of negative treatment are of concern in light of the potential harmful consequences of weight stigma for larger women’s psychosocial wellbeing and health [3,8,33].

Further, women’s pre-pregnancy BMI was not related to perceived quality of treatment during labour and birth. It is possible that for women, during labour, safety and technical proficiency are more salient than is interpersonal treatment from healthcare professionals. Alternatively, weight differences from early pregnancy may be less pronounced in later pregnancy, or at the time of labour and birth, resulting in less difference in quality of treatment according to body size.

Pre-pregnancy BMI was significantly related to perceived positive treatment after birth, but not perceived negative treatment. This suggests that women with a higher BMI perceived less positive treatment overall after birth, compared to lower-BMI women, but were not more likely to experience instances of negative treatment during this time. Perceived risks of obesity in pregnancy [34], and any extra pregnancy management requirements for larger women [32], by their very definition, no longer apply after birth. This may explain the fact that body size did not predict reported negative treatment after birth. Lower perceived positive treatment may reflect a lower general positivity towards women of larger body size among postnatal care providers, possibly on the basis of perceptions that such women are unhealthy [11] or unattractive [35].

Overall, the findings from Study One demonstrated that as BMI increases, women report more negative treatment throughout pregnancy and less positive treatment after giving birth. This contrasts with Hildingsson and Thomas’ [27] finding of no difference in satisfaction with maternity care between women with a BMI below 30 and those with a BMI above 30. However, as noted by the authors, this finding may have related to midwives’ reluctance to discuss weight or diet, in the context of having little training or guidance in Sweden about how to discuss such issues with obese pregnant women. In the Australian context, however, recently-developed clinical guidelines recommend that maternity care professionals discuss weight gain, diet and exercise in pregnancy e.g., [15]. Indeed, the current findings are consistent with a recent large-scale Australian survey that found associations between obesity and perceived discrimination in maternity care [26]. It is important to note, however, that research findings of Study One comprise only women’s *perceptions* of care, and may not be a true reflection of the care provided. Women with a higher BMI are more prone to lower self-esteem and greater depressive symptoms [36], and it is possible that pre-existing differences between groups in mental health may bias women’s perceptions of care and treatment. In light of this possibility, Study Two manipulated the BMI of a hypothetical pregnant patient, and assessed pre-service maternity care providers’ responses.

Study Two found that pre-service maternity care providers responded to hypothetical pregnant women differentially based on their BMI, suggesting that associations between BMI and quality of treatment in Study One are unlikely to be explained (solely) by inaccurate perceptions on the part of women. Firstly, pre-service maternity care providers perceived that pregnant patients with a higher BMI had poorer self-management and overall health. Such findings concur with those of Hebl and Xu [11], who found that general physicians perceived poorer self-management and health among patients with a higher BMI. Importantly, there is no evidence to suggest that overweight and obese patients have poorer self-management behaviours and abilities [37,38]. Accordingly, pre-service maternity care providers’ inaccurate perceptions of differences in self-care between pregnant women of differing body size may negatively impact on their care of pregnant women with a higher BMI.

Pre-service maternity care providers also had less positive attitudes towards caring for overweight and obese pregnant women than normal-weight patients. This result was consistent with evidence from outside the maternity care setting which has shown that healthcare providers have less positive attitudes towards caring for higher-weight than normal-weight patients [11,35]. Pregnant women with large body sizes may be differentially disadvantaged with respect to care providers’ attitudes towards caring for them, negatively affecting the development of warm, sensitive relationships between care providers and higher-BMI pregnant women relative to normal-weight women [11].

In sum, pre-service maternity care providers who read about a normal-weight pregnant woman had more positive perceptions of her self-management and health, and more positive attitudes towards providing care for this patient, than those who read about overweight and obese pregnant women. Interestingly, these relationships were not moderated by general levels of weight stigmatising attitudes. Even care providers with only minor general weight stigmatising attitudes responded less positively to overweight and obese pregnant women compared to normal-weight women. The more participants indicated that they held weight stigmatising attitudes, however, the worse their perceptions of patient self-management and care, and the more negative their attitudes were towards providing care for patients. This unexpected finding suggests that weight stigmatising attitudes may reflect a general negativity bias, and play a role in disrupting patient care, irrespective of patient weight.

Together, our results provide evidence that weight stigma is prevalent in Australian maternity care settings. The observed effects, however, were small. This raises questions about the probable magnitude of disparity between care of normal-weight, overweight and obese pregnant women in clinical practice, and further research is needed to clarify such effects. Nevertheless, it is feasible that our results are only conservative estimates of effects, due to the possible impact of several methodological characteristics. For example, survey data used in Study One was not originally collected for the purpose of investigating weight stigma in maternity care. Items regarding perceived quality of treatment were not developed specifically to tap into differential treatment experienced by pregnant women on the basis of their BMI. Therefore, it is plausible that measures designed specifically to examine weight stigmatising experiences in maternity care may be more sensitive and thus provide stronger evidence for the presence of weight stigma in this context. Additionally, the effects found in Study Two are likely to be conservative estimates of the extent of weight stigma in maternity care. Social desirability bias may have led to under-reporting of both general weight stigma attitudes and negative attitudes towards the patient. Furthermore, we relied on a student sample, and studies have found that healthcare professionals and students with fewer years of working experience display a lower degree of weight-based prejudice than those with more working experience [39,40]. Prolonged exposure to a workplace culture which condones weight stigmatising attitudes, as has been described in qualitative research [41], may inflate weight-based prejudice among health care providers. Future research, both qualitative and quantitative, should determine whether the findings of the current study generalise to clinical settings. It should also assess the impact of differences in care provision on the quality of relationships between maternity care providers and women in practice, as well as on women’s health and other outcomes.

Although the present studies have a number of strengths, including the triangulation of quantitative date from both patients and providers and the inclusion of normal-weight comparison participants, they also contain some methodological limitations. In Study One, sampling via birth notifications with minimal exclusions allowed us to reduce biases associated with sample selection, but the low response rate for this study highlights the importance of careful consideration of potential response bias. Further, the measure of pre-pregnancy BMI in Study One may be prone to recall bias (being asked to report such information from almost a year prior) and social desirability bias. In their systematic review, Gorber, Tremblay, Moher and Gorber [42] found that participants within all weight categories tend to overestimate height and underestimate weight and BMI in self-report, and there is some evidence to suggest that those who are obese under-estimate weight and over-estimate height by greater amounts [43]. In the present study, our analysis of BMI as a continuous variable mitigated against problems of misclassification, though reporting bias may have restricted the range of BMI values. As such, associations between pre-pregnancy BMI and outcome variables are still likely to be valid, though a restricted range of BMI values may have reduced the strength of associations. Stronger relationships between BMI and quality of care may be observed if BMI values are objectively measured rather than self-reported.

The present study was not sufficiently powered to conduct analyses stratified by professional group (midwifery or medical students), and it is possible that the professional group to which one belongs might change or qualify the associations observed. Thus, further research also may seek to investigate differences between professional groups. Additionally, both Study One and Study Two utilised measures which have not been widely used or validated in other research. However, the measure of perceived quality of treatment in Study One was adapted from those used by the UK National Perinatal Epidemiology Unit in their ongoing surveys of maternity care (see [44]). The “perceptions” and “attitudes” variables in Study Two were developed using items from Hebl and Xu [11], who used a similar research design to compare general physicians’ attitudes towards a hypothetical normal-weight versus obese patient. Given the current dearth of research into weight stigma in maternity care, there are no standard questionnaires assessing the constructs we investigated. Thus, future research should seek to identify accurate and useful measures to assess weight stigmatising attitudes and behaviours in maternity care settings.

Overall, the prospect that patient body size alone could influence quality of treatment and care relationships in maternity care, as suggested by the results in Study One, and particularly, Study Two, is of concern. There is a demonstrated detrimental impact of negative weight-related health treatment, and weight stigma more broadly, on psychosocial wellbeing [3,4,33] and health behaviours [5,7]. Such findings should be taken seriously, given the increased vulnerabilities to anxiety, depression and a range of other pressures for women during pregnancy and the postpartum period [18,45]. Notably, findings of Study Two also indicate that a degree of weight-based prejudice manifests among medical and midwifery *students.* Pending further investigation to confirm such effects, this implies that there is a need to address weight stigmatising behaviours, attitudes, and beliefs in these formative years of study. Further, findings also indicate that a patient’s body size has an independent effect on students’ attitudes, regardless of general weight stigmatising attitudes. Even students with low levels of general weight stigma attitudes may automatically respond more negatively to overweight and obese patients, highlighting the need for further work to determine the underlying mechanisms for differential treatment of patients on the basis of body size. Finally, future research should expand its focus to investigate the occurrence of any differential or stigmatising treatment of under-weight women in maternity care as well. There is a growing focus on risks and management of underweight women in pregnancy e.g., [46,47], yet a lack of information about experienced stigma among this group.

Greater frustration with the management of obese pregnant patients [32], lower perceived confidence and knowledge regarding obesity [48], and perceptions of higher risks [34] may all contribute to weight stigma among maternity care providers. Such factors may also be exacerbated by a working environment with a lack of appropriate resources and adequate support for maternity care professionals, negatively impacting on their ability to properly support and care for pregnant patients with a higher BMI. Furness et al. [21] found that midwives often felt uncomfortable raising weight-related issues with women, but that women attending a clinic with specialist midwives (with additional training and experience working with pregnant women with a higher BMI) benefited from clear, non-judgmental advice and support about issues related to their weight and pregnancy. Greater support and training from specialist midwives, and/or clear referral pathways to specialist midwives, may assist less experienced midwives in providing better care for women with a higher BMI. Accordingly, further research investigating the systemic barriers to providing individualized, non-judgmental high quality care for obese women, and the effectiveness of strategies to overcome them, including from providers’ perspectives, is required.

## Conclusions

These studies used population data and an experimental design to provide preliminary evidence for weight stigma in Australian maternity care from the perspective of care recipients and providers, respectively. Our findings provide sufficient basis for further research to uncover contributing factors to weight stigmatising attitudes and behaviours among maternity care providers. As the factors contributing to weight stigma become clearer, effective ways to target such factors to reduce weight stigmatising behaviour should be investigated and implemented, both in clinical settings and in medical and midwifery university training programs (see [22] for an example of an anti-weight bias intervention developed for health professionals in training). It is of great importance that any experienced disadvantage or inequality among overweight and obese women in maternity care is removed. All women, regardless of body size, deserve equal access to safe and supportive maternity care, and equal opportunities to experience full enjoyment of such a significant life event.

## Endnotes

^a^Pre-service maternity care providers refers to students in their final year of a three-year Midwifery degree, or students in their final two years of either a four-year graduate medical degree or a six-year undergraduate medical degree. In Australia, some universities offer a four-year graduate medical degree which students may apply to after the completion of an undergraduate program, whereas others offer an undergraduate medical degree which students may apply to upon completing school. Both medical and midwifery students obtain experience in hospitals and other clinical settings from the first year of their program, with medical students completing more intensive clinical rotations in hospitals in their final two years. General medical practitioners, obstetricians and midwives are the main providers of maternity care in Australia, hence the focus on pre-service care providers in these two disciplines in this study.

^b^A General Practice rotation within a Medical Degree usually involves training and experience in pregnancy/antenatal care, and typically lasts 8 to 10 weeks.

## Competing interests

The authors have no competing interests to declare.

## Authors’ contributions

KM designed the studies, collected the data for study 2, conducted the statistical analysis and interpretation of results and drafted the manuscript. YM conceived the studies, participated in the design for Study 1 and Study 2, designed the measures and collected the data for Study 1, and participated in writing of the manuscript. FKB participated in the design of the studies, design of measures for Study 2, consulted on the statistical analysis, interpreted the results and participated in drafting the manuscript. PD participated in the design of Study 2, interpretation of the results and writing of the manuscript. RT contributed to the conception of the study, participated in the design for Study 1 and Study 2, designed the measures and collected the data for Study 1, and participated in writing of the manuscript. All authors read and approved the final manuscript.

## Pre-publication history

The pre-publication history for this paper can be accessed here:

http://www.biomedcentral.com/1471-2393/13/19/prepub
